# Importation of Hybrid Human-Associated *Trypanosoma cruzi* Strains of Southern South American Origin, Colombia

**DOI:** 10.3201/eid2208.150786

**Published:** 2016-08

**Authors:** Louisa A. Messenger, Juan David Ramirez, Martin S. Llewellyn, Felipe Guhl, Michael A. Miles

**Affiliations:** London School of Hygiene and Tropical Medicine, London, UK (L.A. Messenger, M.S. Llewellyn, M.A. Miles);; Universidad del Rosario, Bogotá, Colombia (J.D. Ramirez); Universidad de Los Andes, Bogotá (F. Guhl)

**Keywords:** Chagas disease, Colombia, South America, Trypanosoma cruzi, characterization, hybridization, TcV, TcVI, clones, hybrid clones, parasites, humans, geographic distribution, ecologic distribution, genotyping, high-resolution nuclear genotyping, high-resolution mitochondrial genotyping, interlineage hybrids, vector-borne infections, discrete typing unit

## Abstract

We report the characterization of *Trypanosoma cruzi* of southern South American origin among humans, domestic vectors, and peridomestic hosts in Colombia using high-resolution nuclear and mitochondrial genotyping. Expanding our understanding of the geographic range of lineage TcVI, which is associated with severe Chagas disease, will help clarify risk of human infection for improved disease control.

Chagas disease is the most common parasitic infection in Latin America, annually affecting ≈5–6 million persons and putting another 70 million at risk (*1*). The etiologic agent, *Trypanosoma cruzi*, displays remarkable genetic diversity, which is widely thought to contribute to the considerable biologic, epidemiologic, and clinical variation observed in regions where the disease is endemic (*2*). Seven discrete typing units (DTUs) are currently recognized (TcI–TcVI and TcBat) (*2*); TcV and TcVI are natural interlineage hybrids of TcII and TcIII (*3*). It is unknown whether these hybrids arose from multiple independent recombination events (*3*) or a single incidence of hybridization followed by clonal divergence (*4*). Molecular dating indicates these lineages evolved recently (<1 million years ago) (*3*,*4*), suggesting that genetic exchange may still be driving the emergence of novel recombinants (*3*,*4*).

Historically, most *T. cruzi* DTUs have had broadly distinct, but often overlapping, geographic and ecologic distributions (*2*). TcV and TcVI are largely confined to domestic transmission cycles and are sympatric with severe chronic and congenital human disease in southern South America (*2*). Increased sampling indicates that the geographic ranges of TcV and TcVI are more extensive than previously suggested. Putative domestic hybrid strains were identified recently as far north as Colombia (*5*); it is unclear whether these are bona fide TcV and TcVI isolates (suggesting long-range introduction) or progeny of a novel, independent, and local recombination event(s). Elucidation of the molecular epidemiology of TcV and TcVI has been complicated by limited sample collections and difficulties distinguishing these genotypes from their parental DTUs (*6*) and each other (*7*). We undertook high-resolution nuclear and mitochondrial genotyping of hybrid clones from Colombia to resolve their putative status as novel recombinants and provide further insights into the evolutionary origin(s) of TcV and TcVI.

## The Study

**Figure 1:**
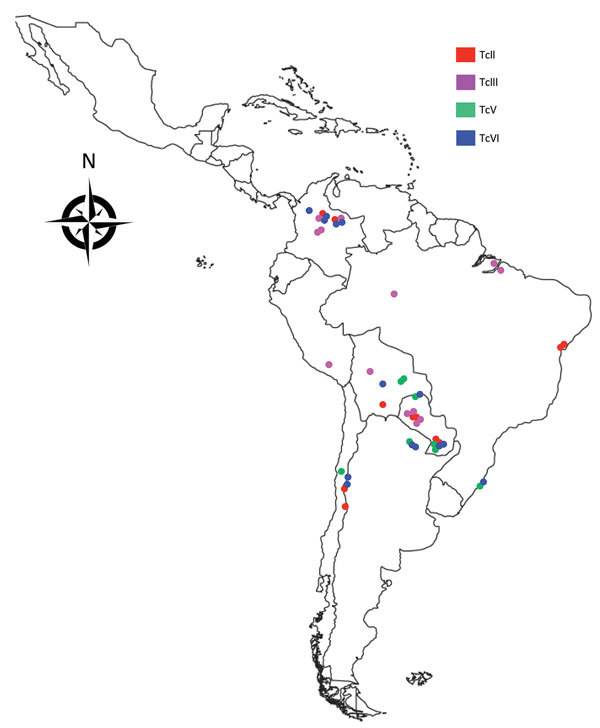
Geographic distribution of TcII, TcIII, TcV, and TcVI *Trypanosoma cruzi* clones, South America, 2002–2010. A total of 57 *T. cruzi* biologic clones were assembled for analysis. Of these, 24 were isolated from humans; triatomine vectors (*Panstrongylus geniculatus, Rhodnius prolixus*, and *Triatoma venosa* insects); and sylvatic mammalian hosts (*Dasypus* spp. armadillos) in Antioquia, Boyaca, and Casanare Departments in northern Colombia. The remaining 33 were reference clones derived from a range of representative hosts and vectors across South America (Technical Appendix 1 Table 1). Dots indicate geographic strain origin of biologic clones; colors denote isolate discrete typing units.

For analysis, we assembled a panel of 57 *T. cruzi* biologic clones from a range of representative hosts/vectors across South America: 24 uncharacterized clones from Colombia and 33 reference clones (Figure 1; Technical Appendix 1 Table 1). From 2002–2010, we isolated the uncharacterized clones from humans; triatomine vectors (*Panstrongylus geniculatus, Rhodnius prolixus*, and *Triatoma venosa* insects); and sylvatic mammalian hosts (*Dasypus* spp. armadillos) in 3 *T. cruzi*–endemic departments in northern Colombia.

We genotyped all isolates using nuclear housekeeping genes *GPX*, *GTP*, *Met-II*, *TcAPX,* and *TcMPX* (*6*,*8*) (online Technical Appendix 1 Table 2); 25 microsatellite loci (Technical Appendix 1 Table 3) (*9*); and 10 mitochondrial gene fragments (*10*). Diploid multilocus sequence typing (MLST) data were analyzed by locus and concatenated according to their relative chromosomal positions in MLSTest (*11*); heterozygous variable sites were handled as average states. Gene haplotypes were inferred using PHASE version 2.1 (*12*). PCR products were cloned and sequenced to confirm ambiguous gene phases. We constructed maximum-likelihood and Bayesian phylogenies for nuclear haplotypic and concatenated mitochondrial data (*13*).

**Figure 2:**
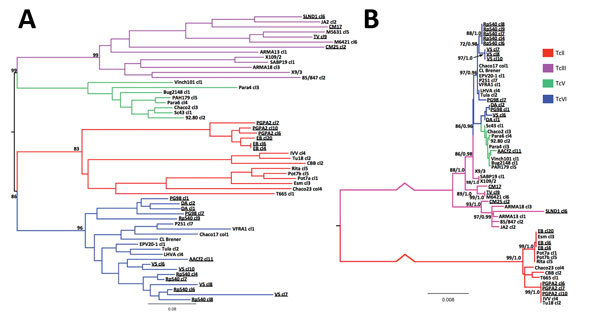
Phylogenetic trees showing relationships between *Trypanosoma cruzi* hybrids from Colombia and reference *T. cruzi* strains from across South America. A) Unrooted neighbor-joining tree based on pairwise distances between microsatellite loci. B) Maximum-likelihood tree from concatenated maxicircle sequences. Pairwise distance–based bootstrap values were calculated as the mean across 1,000 random diploid resamplings of the dataset; those >70% are shown for relevant nodes. A maximum-likelihood topology was constructed from concatenated maxicircle sequences for all clones. The most appropriate nucleotide substitution model was the general time reversible plus gamma distribution (9 substitution rate categories) based on the Akaike information criterion. Statistical support for major clades is given as equivalent bootstraps and posterior probabilities from consensus maximum-likelihood (1,000 pseudo-replicates) and Bayesian trees (based on the Hasegawa-Kishino-Yano plus gamma distribution model), respectively. Note that strain AACf2 cl11 is phylogenetically incongruent between nuclear and mitochondrial topologies. Branch colors indicate isolate discrete typing unit. Labels for clones from Colombia are underlined. Scale bars indicate genetic distance (A) and nucleotide substitutions per site (B).

For microsatellite loci, we defined sample clustering using a neighbor-joining tree based on pairwise distances between multilocus genotypes (Figure 2) (*13*). We calculated DTU-level heterozygosity (Bonferroni-corrected) and evaluated genetic diversity using sample size–corrected allelic richness and private allele frequency per locus (Table). To examine TcV/TcVI allele inheritance, we classified genotypes at each locus as hybrid (TcII/TcIII) or nonhybrid (TcII/TcII or TcIII/TcIII) based on the presence or absence of specific parental alleles (Technical Appendix 2).

**Table:** Population genetic parameters for *Trypanosoma cruzi* discrete typing units, South America, 2002–2010*

Discrete typing unit	No. multilocus genotypes/no. isolates	Proportion shared alleles ± SD	No. polymorphic loci	Mean no. private alleles per locus ± SE	Mean A_r_ ± SE†	Mean expected/observed heterozygosity†	% Loci with deficit/excess heterozygosity‡
TcII	14/15 (5/6)	0.44 ± 0.23 (0.062 ± 0.053)	24 (15)	1.76 ± 0.20 (0.68 ± 0.14)	3.94 ± 0.29 (1.65 ± 0.12)	0.58/0.65 (0.91/0.58)	29.2/20.8 (40.0/0)
TcIII	13/13 (4/4)	0.48 ± 0.15 (0.30 ± 0.16)	22 (21)	2.35 ± 0.48 (1.76 ± 0.27)	4.26 ± 0.43 (2.35 ± 0.18)	0.45/0.70 (0.46/0.69)	4.5/27.3 (9.5/38.1)
TcV	8/8	0.15 ± 0.092	22	0.16 ± 0.07	2.38 ± 0.20	0.85/0.58	54.6/4.5
TcVI	21/21 (14/14)	0.24 ± 0.87 (0.22 ± 0.103)	21 (20)	0.43 ± 0.12 (0.86 ± 0.20)	2.46 ± 0.21 (1.87 ± 0.11)	0.60/0.49 (0.71/0.54)	41.7/16.7 (40.0/15.0)

*Values represent findings for reference clones derived from a range of representative hosts and vectors across South America and, in parentheses, clones isolated from humans, triatomine vectors, and sylvatic mammalian hosts in northern Colombia. Values were calculated from microsatellite data for 25 analyzed loci. Ar, allelic richness.  †Across all loci. ‡After sequential Bonferroni correction.

All putative hybrids from Colombia were highly heterozygous and minimally diverse. They possessed TcII and TcIII alleles at an approximate 1:1 ratio and, compared with parental DTUs, they displayed fewer private alleles or single-nucleotide polymorphisms; these strains fulfilled all the expectations of progeny from a recent Mendelian hybridization event(s) (Table). Based on nuclear MLST and microsatellite data, all hybrids from Colombia were classified as TcVI, not novel recombinants.

Examination of TcII and TcIII alleles across 5 nuclear loci showed that hybrid haplotypes from Colombia were shared among other TcVI strains from the Southern Cone region of South America and showed negligible affinities to parental alleles from Colombia (Technical Appendix 1 Figures 1, 2). Microsatellite profiles also supported this allopatric inheritance: only a minority of private parental alleles from Colombia were common to local TcVI hybrids. At mitochondrial loci, TcVI clones from Colombia were noticeably divergent from local TcIII maxicircle haplotypes and those observed in reference TcVI strains (Figure 2). Of note, 1 hybrid from Colombia (AACf2 cl11), which was unequivocally classified as TcVI by both types of nuclear loci, possessed a TcV-type mitochondria. All isolates in this study were biologic clones, ruling out mixed infections as a potential confounder.

Overall, our data support the hypothesis that 2 separate recombination events led to the formation of TcV and TcVI. These interlineage hybrids have distinct nuclear and mitochondrial MLST genotypes and related but independent microsatellite profiles, and most alleles that distinguish between hybrid DTUs (70.4% [38/54 alleles]) were also present in their corresponding parental strains. Interlineage differences (fixed at 84% [21/25 of loci]) between TcV and TcVI are not consistent with allelic sequence divergence (Meselson effect); for such divergence, a much higher frequency of private alleles, compared with parental genotypes, would be expected at rapidly evolving microsatellite loci.

TcVI clones from Colombia had more private microsatellite alleles per locus (0.86) than their southern counterparts (0.43), despite their unequivocal origin in the Southern Cone. This phenomenon could be attributable to de novo mutations or a founder effect with respect to the northerly introduction of TcVI. Support for the latter cause is evidenced by an overall reduction in genetic diversity among hybrids from Colombia compared with TcVI strains from the Southern Cone (allelic richness 1.87 vs. 2.46, respectively). However, we cannot discount some sampling bias because reference Southern Cone strains represented a much wider geographic range.

A novel observation among TcVI strains from Colombia was the presence of an anomalous TcV maxicircle. This pattern of inheritance could reflect 1) recent mitochondrial introgression from TcV into TcVI, leaving undetectable signatures of nuclear hybridization by our markers or, possibly, none at all (*10*,*14*), or 2) potential backcrossing of TcVI into TcIII. Genetic exchange has not been described among hybrid DTUs, but it might be expected to be more permissive between closely related strains (*14*). We also isolated hybrid AACf2 cl11 from a dog. *T. cruzi* hybridization has been proposed to arise within mammalian cells (*14*), and mixed infections in such hosts are common. Alternatively, TcV and TcVI may have evolved from the beneficiaries of different alleles during a single hybridization event between heterozygous parents with mixed TcIII-type mitochondrial complements; although, to date, reported levels of mitochondrial heteroplasmy in *T. cruzi* are low (*10*).

## Conclusions

Our understanding of the geographic and ecologic distribution of *T. cruzi* DTUs is changing because of parallel improvements in sampling strategies and genotyping techniques. Human Chagas disease in Colombia is currently associated with DTUs TcI, TcII (to a lesser extent), and oral outbreaks of TcIV (*5*). In this study, we isolated* T. cruzi* hybrids from 3 domestic triatomine vectors, a peridomestic dog, and congenital infections among local patients. Given that no reservoir hosts of TcV and TcVI have been described (*15*), the hybrids from Colombia are more likely the result of long-range anthropogenic introduction than local sylvatic invasion, especially considering the successful establishment of these DTUs among domestic infections in the Southern Cone. Further intensive sampling efforts in northern South America are warranted to elucidate the transmission cycle ecology of TcVI and to accurately assess the epidemiologic risk of human Chagas disease associated with this low-diversity hybrid lineage.

Technical Appendix 1 Strain origins, nuclear and mitochondrial MLST analyses, and microsatellite loci and primer information for *Trypanosoma cruzi* clones from Colombia and reference clones from across South America.

Technical Appendix 2 Microsatellite allele profiles used to determine inheritance patterns among *Trypanosoma cruzi *clones from Colombia and reference clones from across South America.
