# Supplementary material for: Importation of Hybrid Human-Associated Trypanosoma cruzi Strains of Southern South American Origin, Colombia
Source: Emerg Infect Dis. 2016 Aug;22(8):1452–5. doi: 10.3201/eid2208.150786 (PMC4982185; doi:10.3201/eid2208.150786)
Supplement: Technical Appendix 1 — Strain origins, nuclear and mitochondrial MLST analyses, and microsatellite loci and primer information for Trypanosoma cruzi clones from Colombia and reference clones from across South America. [file 15-0786-Techapp-s1.pdf]

Article DOI: http://dx.doi.org/10.3201/eid2208.150786

Importation of Hybrid Human-Associated *Trypanosoma cruzi* Strains of Southern South American Origin, Colombia

Technical Appendix 1

Technical Appendix 1 Table 1. Panel of Colombian biologic clones and reference clones assembled for analysis.

Strain code	Host/vector	Department	Country*	Discrete typing unit
Colombian Clones†				
EB cl4‡	*Homo sapiens* neonate (suspected congenital infection)	Boyaca	Colombia	TcII
EB cl6	*Homo sapiens* neonate (suspected congenital infection)	Boyaca	Colombia	TcII
EB cl20	*Homo sapiens* neonate (suspected congenital infection)	Boyaca	Colombia	TcII
PGPA2 cl6	*Panstrongylus geniculatus*	Casanare	Colombia	TcII
PGPA2 cl7	*Panstrongylus geniculatus*	Casanare	Colombia	TcII
PGPA2 cl10	*Panstrongylus geniculatus*	Casanare	Colombia	TcII
CM17	*Dasypus sp.*	Carimagua	Colombia	TcIII
CM25 cl2	*Dasypus novemcinctus*	Carimagua	Colombia	TcIII
SLDN1 cl6	*Dasypus novemcinctus*	Casanare	Colombia	TcIII
TV cl9	*Triatoma venosa*	Boyaca	Colombia	TcIII
AACf2 cl11	*Canis familiaris*	Casanare	Colombia	TcVI
DA cl1	*Homo sapiens* adult (suspected congenital transmitter)	Boyaca	Colombia	TcVI
DA cl2	*Homo sapiens* adult (suspected congenital transmitter)	Boyaca	Colombia	TcVI
PG98 cl1	*Panstrongylus geniculatus*	Antioquia	Colombia	TcVI
PG98 cl7	*Panstrongylus geniculatus*	Antioquia	Colombia	TcVI
Rp540 cl4	*Rhodnius prolixus*	Casanare	Colombia	TcVI
Rp540 cl6	*Rhodnius prolixus*	Casanare	Colombia	TcVI
Rp540 cl7	*Rhodnius prolixus*	Casanare	Colombia	TcVI
Rp540 cl8	*Rhodnius prolixus*	Casanare	Colombia	TcVI
Rp540 cl9	*Rhodnius prolixus*	Casanare	Colombia	TcVI
VS cl6	*Homo sapiens* neonate (suspected congenital infection)	Boyaca	Colombia	TcVI
VS cl7	*Homo sapiens* neonate (suspected congenital infection)	Boyaca	Colombia	TcVI
VS cl8	*Homo sapiens* neonate (suspected congenital infection)	Boyaca	Colombia	TcVI
VS cl10	*Homo sapiens* neonate (suspected congenital infection)	Boyaca	Colombia	TcVI
Reference Clones§				
CBB cl2	*Homo sapiens*	Tulahuén	Chile	TcII
Chaco23 col4	*Triatoma infestans*	Pr. Hayes	Paraguay	TcII
Esm cl3	*Homo sapiens*	*São Felipe*	Brazil	TcII
IVV cl4	*Homo sapiens*	Cuncumen	Chile	TcII
Pot7a cl1	*Triatoma infestans*	San Martin	Paraguay	TcII
Pot7b cl5	*Triatoma infestans*	San Martin	Paraguay	TcII

Strain code	Host/vector	Department	Country*	Discrete typing unit
Rita cl5	*Homo sapiens*	*São Felipe*	Brazil	TcII
T665 cl1	*Triatoma infestans*	Pr. Hayes	Paraguay	TcII
Tu18 cl2	*Triatoma infestans*	Tupiza	Bolivia	TcII
85/847 cl2	*Dasypus novemcinctus*	Alto Beni	Bolivia	TcIII
ARMA13 cl1	*Dasypus novemcinctus*	Campo Lorro	Paraguay	TcIII
ARMA18 cl3	*Dasypus novemcinctus*	Campo Lorro	Paraguay	TcIII
JA2 cl2	*Monodelphis sp.*	Amazonas	Brazil	TcIII
M5631 cl5	*Dasypus novemcinctus*	Marajo	Brazil	TcIII
M6421 cl6	*Homo sapiens*	Belém	Brazil	TcIII
SABP19 cl1	*Triatoma infestans*	Vitor	Peru	TcIII
X109/2	*Canis familiaris*	Makthlawaiya	Paraguay	TcIII
X9/3	*Canis familiaris*	Makthlawaiya	Paraguay	TcIII
92.80 cl2	*Homo sapiens*	Santa Cruz	Bolivia	TcV
Bug 2148 cl1	*Triatoma infestans*	Rio Grande do Sul	Brazil	TcV
Chaco2 cl3	*Triatoma infestans*	Chaco	Paraguay	TcV
PAH179 cl5	*Homo sapiens*	Chaco	Argentina	TcV
Para4 cl3	*Triatoma infestans*	Paraguari	Paraguay	TcV
Para6 cl4	*Triatoma infestans*	Paraguari	Paraguay	TcV
Sc43 cl1	*Triatoma infestans*	Santa Cruz	Bolivia	TcV
Vinch101 cl1	*Triatoma infestans*	Limari	Chile	TcV
Chaco17 col1	*Triatoma infestans*	Chaco	Paraguay	TcVI
CL Brener	*Triatoma infestans*	Rio Grande do Sul	Brazil	TcVI
EPV20–1 cl1	*Triatoma infestans*	Chaco	Argentina	TcVI
LHVA cl4	*Triatoma infestans*	Chaco	Argentina	TcVI
P251 cl7	*Homo sapiens*	Cochabamba	Bolivia	TcVI
Tula cl2	*Homo sapiens*	Tulahuén	Chile	TcVI
VFRA1 cl1	*Triatoma infestans*	Francia	Chile	TcVI

*References (*1*–*5*) describe the different geographic distributions, host/vector associations, and transmission cycles of *T. cruzi* DTUs in Colombia.
†Colombian clones were assigned to DTU-level by PCR amplification of the *SL-IR, 24α rDNA* and *18S rDNA* subunits according to (*6*). Putative hybrid strains were identified by either a double *24α rDNA* amplicon (125 and 140 bp) (TcV) or single *24α rDNA* amplicon (140 bp) and amplification of the A10 fragment of the *18S rDNA* subunit (TcVI) (525 or 630 bp), and confirmed by sequencing glucose-6-phosphate isomerase (*GPI*), as previously described (*6*).
‡Indicates multiple biologic clones derived from a single parasite strain.
§Reference clones were assigned to DTU-level using a triple-marker assay described by Lewis et al. (*7*).

Technical Appendix 1 Table 2. Intra-lineage diversity and properties of nuclear and mitochondrial MLST schemes*

		Housekeeping gene																											
		GPX				*GTP*				*Met-II*				*TcAPX*				*TcMPX*				nMLST†				mtMLST‡			
T. cruzi DTU	Total no. isolates	VS	ST	TE	DP	VS	ST	TE	DP	VS	ST	TE	DP	VS	ST	TE	DP	VS	ST	TE	DP	VS	ST	TE	DP	VS	ST	TE	DP
TcII	15 [6]	4 [0]	6 [1]	1.5 [0]	0.4 [0.17]	2 [0]	3 [1]	1.5 [0]	0.2 [0.17]	5 [0]	6 [1]	1.2 [0]	0.4 [0.17]	2 [0]	3 [1]	1.5 [0]	0.2 [0.17]	8 [0]	4 [1]	0.5 [0]	0.27 [0.17]	21 [0]	10 [1]	0.48 [0]	0.67 [0.17]	46 [25]	7 [3]	0.15 [0.12]	0.47 [0.5]
TcIII	13 [4]	10 [4]	8 [3]	0.8 [0.75]	0.62 [0.75]	2 [1]	3 [2]	1.5 [2.0]	0.23 [0.5]	10 [5]	7 [3]	0.7 [0.6]	0.54 [0.75]	4 [3]	5 [3]	1.25 [1.0]	0.38 [0.75]	1 [1]	3 [2]	3.0 [2.0]	0.23 [0.5]	27 [13]	13 [4]	0.48 [0.31]	1.0 [1.0]	107 [80]	10 [4]	0.093 [0.05]	0.77 [1.0]
TcV	8 [0]	0	1	0	0.125	0	1	0	0.125	17	2	0.12	0.25	9	4	0.44	0.5	5	2	0.4	0.25	31	5	0.16	0.63	6	8	1.33	1
TcVI	21 [14]	10 [2]	4 [3]	0.4 [1.5]	0.19 [0.21]	5 [5]	3 [3]	0.6 [0.6]	0.14 [0.21]	14 [0]	4 [1]	0.29 [0]	0.19 [0.07]	11 [11]	7 [4]	0.64 [0.36]	0.33 [0.29]	5 [0]	5 [1]	1.0 [0]	0.24 [0.07]	42 [12]	16 [9]	0.38 [0.75]	0.76 [0.86]	26 [26]	9 [7]	0.35 [0.27]	0.43 [0.5]

*Nos. in square brackets represent strains from Columbia. DP, no. of genotypes identified per total no. of isolates; DTU, discrete typing unit; MLST, multilocus sequence typing; mtMLST, mitochondrial MLST scheme, nMLST, nuclear MLST scheme, ST, no. of genotypes; TE, no. of genotypes identified per polymorphic site; VS, no. of variable sites.
†Based on 5 concatenated loci.
‡Based on 10 concatenated loci.

Technical Appendix 1 Table 3. Panel of microsatellite loci and primers employed in this study*

Chromosome	Primer code	Repeat type	Forward/reverse primer (5′→3′)
6	6529(CA)$_a$	(CA)$_n$	TGTGAAATGATTTGACCCGA<br>AGAGTCACGCCGCAAAGTAT
6	6529(TA)$_b$	(TA)$_n$	TGAAGGAGATTCTCTGCGGT<br>CTCTCATCTTTTGTTGTGTCCG
6	mclf10	(CA)$_n$A(CA)$_n$	GCGTAGCGATTCATTTCC<br>ATCCGCTACCACTATCCAC
10	6855(TA)(GA)	(TA)$_n$(GA)$_n$	TGTGATCAACGCGCATAAAT<br>TTCCATTGCCTCGTTTTAGA
15	11863(CA)	(CA)$_n$	AGTTGACATCCCCAAGCAAG<br>CCCTGATGCTGCAGACTCTT
19	10101(TA)	(TA)$_n$	AACCCGCGCAGATACATTAG<br>TTCATTTGCAGCAACACACA
24	8741(TA)	(TA)$_n$	TGTAACGGTAGGTCTCAATTCG<br>TTGCACTTGTGTATCTCGCC
27	10101(TC)	(TC)$_n$	CGTACGACGTGGACACAAAC<br>ACAAGTGGGTGAGCCAAAAG
27	10101(CA)$_c$	(CA)$_n$	GTGTCGTTGCTCCCAAACTC<br>AAACTTGCCAAATGTGAGGG
27	10101(CA)$_a$	(CA)$_n$	GTCGCCATCATGTACAAACG<br>CTGTTGGCGAATGGTCATAA
34	6559(TC)	(TC)$_n$	CGCTCTCAAAGGCACCTTAC<br>ATATGGACGCGTAGGAGTGC
37	10187(TTA)	(TTA)$_n$	GAGAGAGATTCGGAAACTAATAGC<br>CATGTCCCTTCCTCCGTAAA
37	10187(CA)(TA)	(CA)$_n$(TA)$_n$	CATGTCATTAAGTGGCCACG<br>GCACATGTTGGTTGTTGGAA
37	10187(TA)	(TA)$_n$	AGAAAAAGGTTTACAACGAGCG

Chromosome	Primer code	Repeat type	Forward/reverse primer (5′→3′)
			CGATGGAGAACGTGAAACAA
37	10187(GA)	$(GA)_n$	GTCACACCACTAGCGATGACA<br>ACTGCACAATACCCCCTTTG
37	TcUn4	Unknown	ATGCTCCGCAACATATTACTCA<br>GTCGAGCTTCTGTTGTTCCC
39	$6925(TG)_b$	$(TG)_n$	GAAACGCACTCACCCACAC<br>GGTAGCAACGCCAAACTTTC
39	7093(TC)	$(TC)_n$	CCAACATTCAACAAGGGAAA<br>GCATGAATATTGCCGGATCT
39	6925(CT)	$(CT)_n$	CATCAAGGAAAAACGGAGGA<br>CGGTACCACCTCAAGGAAAG
39	$7093(TA)_c$	$(TA)_n$	CGTGTGCACAGGAGAGAAAA<br>CGTTTGGAGGAGGATTGAGA
39	$6925(TG)_a$	$(TG)_n$	TCGTTCTCTTTACGCTTGCA<br>TAGCAGCACCAAACAAAACG
39	7093(TCC)	$(TCC)_n$	AGACGTTCATATTCGCAGCC<br>AGCCACATCCACATTTCCTC
40	11283(TCG)	$(TCG)_n$	ACCACCAGGAGGACATGAAG<br>TGTACACGGAACAGCGAAG
40	$11283(TA)_b$	$(TA)_n$	AACATCCTCCACCTCACAGG<br>TTTGAATGCGAGGTGGTACA
41	10359(CA)(GA)	$(CA)_n(GA)_n$	AGTCCTACTGCCTCCTTGCA<br>CTGTTGGCGAATGGTCATAA

*A possible confounder that must be considered during data interpretation is that due to the high mutation rate of microsatellites, potentially as high as 1/1,000 cell divisions (*8*), and between different loci, some of the length variation observed may be de novo, arising during parasite isolation/culturing, not during natural strain evolution and transmission.

References

1. Ramírez JD, Turriago B, Tapia-Calle G, Guhl F. Understanding the role of dogs (*Canis lupus familiaris*) in the transmission dynamics of *Trypanosoma cruzi* genotypes in Colombia. Vet Parasitol. 2013;196:216–9. PubMed http://dx.doi.org/10.1016/j.vetpar.2012.12.054

2. Ramírez JD, Guhl F, Rendon LM, Rosas F, Marin-Neto JA, Morillo CA. Chagas cardiomyopathy manifestations and *Trypanosoma cruzi* genotypes circulating in chronic Chagasic patients. PLoS Negl Trop Dis. 2010;4:e899. PubMed http://dx.doi.org/10.1371/journal.pntd.0000899

3. Zafra G, Mantilla JC, Valadares HM, Macedo AM, Gonzalez CI. Evidence of *Trypanosoma cruzi* II infection in Colombian chagasic patients. Parasitol Res. 2008;103:731–4. PubMed http://dx.doi.org/10.1007/s00436-008-1034-0

4. Mantilla JC, Zafra GA, Macedo AM, Gonzalez CI. Mixed infection of *Trypanosoma cruzi* I and II in a Colombian cardiomyopathic patient. Hum Pathol. 2010;41:610–3. PubMed http://dx.doi.org/10.1016/j.humpath.2009.11.005

5. Ramírez JD, Montilla M, Cucunubá ZM, Floréz AC, Zambrano P, Guhl F. Molecular epidemiology of human oral Chagas disease outbreaks in Colombia. PLoS Negl Trop Dis. 2013;7:e2041. PubMed http://dx.doi.org/10.1371/journal.pntd.0002041

6. Guhl F, Ramírez JD. Retrospective molecular integrated epidemiology of Chagas disease in Colombia. Infect Genet Evol. 2013;20:148–54. PubMed http://dx.doi.org/10.1016/j.meegid.2013.08.028

7. Lewis MD, Ma J, Yeo M, Carrasco HJ, Llewellyn MS, Miles MA. Genotyping of *Trypanosoma cruzi*: systematic selection of assays allowing rapid and accurate discrimination of all known lineages. Am J Trop Med Hyg. 2009;81:1041–9. PubMed http://dx.doi.org/10.4269/ajtmh.2009.09-0305

8. Brinkmann B, Klintschar M, Neuhuber F, Huhne J, Rolf B. Mutation rate in human microsatellites: influence of the structure and length of the tandem repeat. Am J Hum Genet. 1998;62:1408–15. PubMed http://dx.doi.org/10.1086/301869

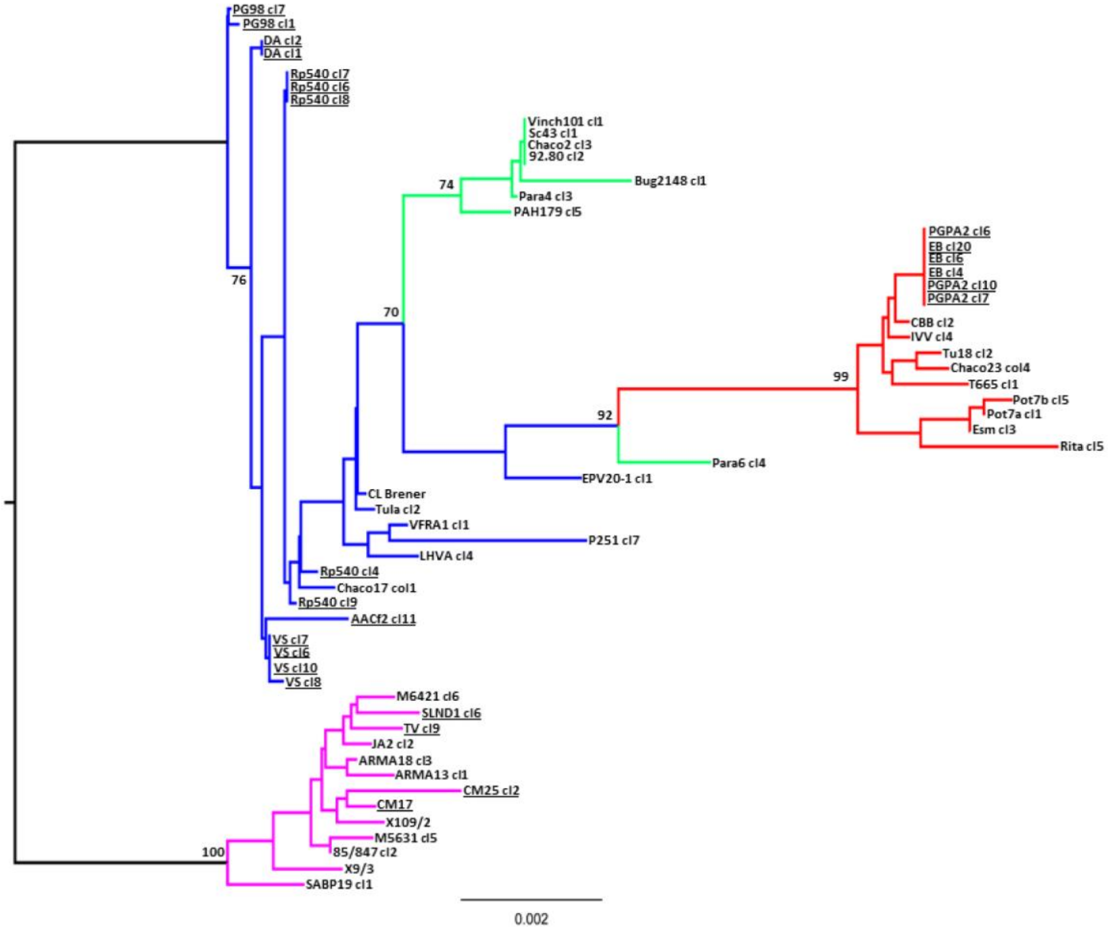

Technical Appendix Figure 1. Unrooted Neighbor-Joining tree based on five concatenated diploid nuclear MLST sequences. For each isolate, nuclear diploid sequence data were concatenated in order of their relative chromosomal positions (Met-II, GTP, TcMPX, TcGPX and TcAPX, on chromosomes 6, 12, 22, 35 and 36, respectively). In MLSTest, phylogenetic incongruence between loci was assessed using the BIO-Neighbor Joining Incongruence Length Difference test (BIONJ-ILD) and evaluated by a permutation test with 1,000 replicates. A final Neighbor-Joining tree was constructed and statistical support was calculated as the mean across 1,000 randomizations and those >70% are shown for relevant nodes. Branch colors indicate isolate DTU (TcII, TcIII, TcV or TcVI). Colombian strain labels are underlined.

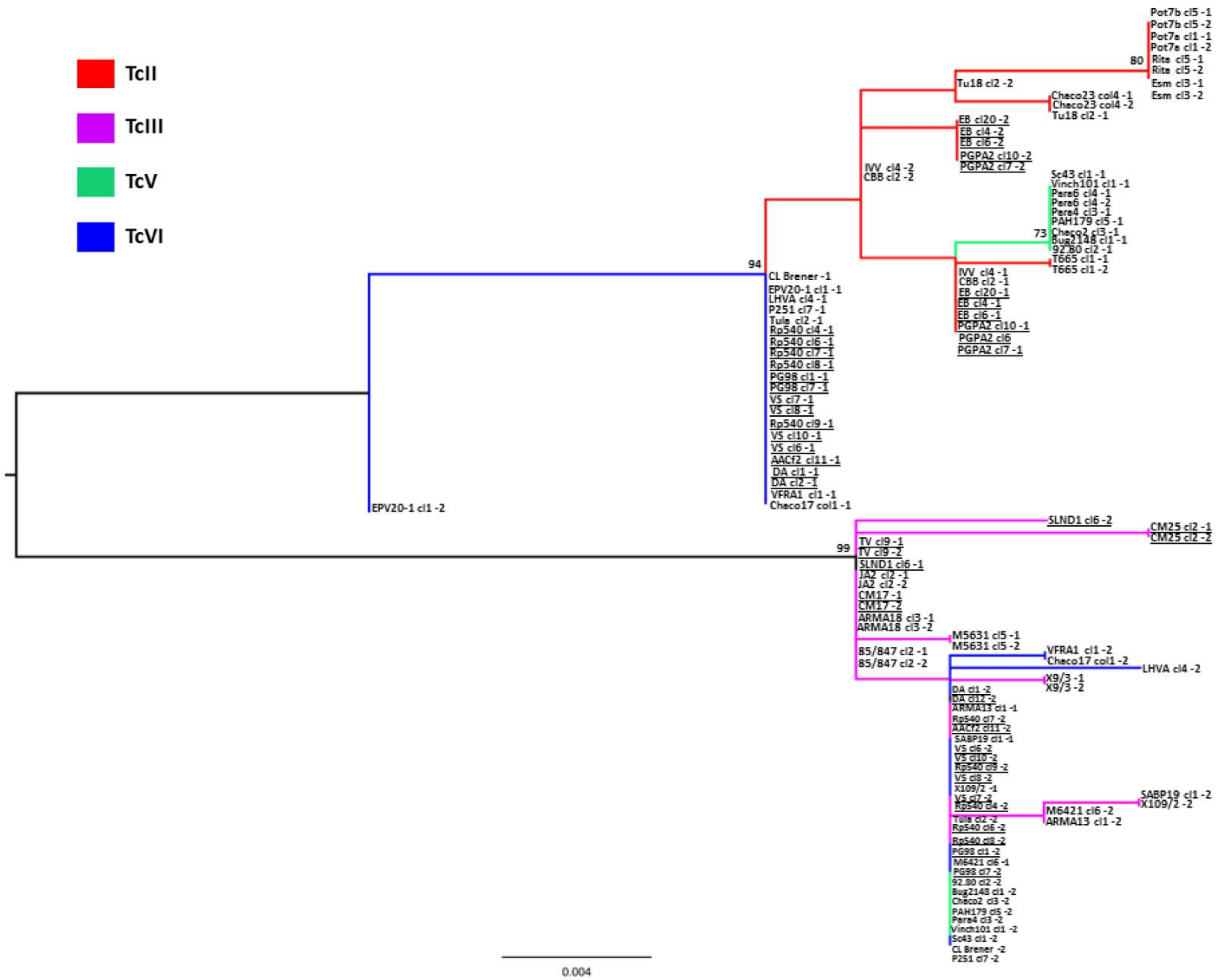

Technical Appendix Figure 2. Maximum-Likelihood tree constructed from Met-II haplotypes. Haplotypes for each nuclear gene were inferred using PHASE v2.1 software, which utilizes a modified Markov chain Monte Carlo (MCMC) algorithm to identify all unambiguous haplotypes within a population, i.e., those observed in strains which are homozygous at all variable sites or heterozygous at only a single polymorphic site.

Haplotypes in the remaining isolates, which are heterozygous at multiple sites (and therefore of ambiguous phase), are then estimated and a probability of uncertainty assigned to each phase call (latterly confirmed by PCR cloning if $p<0.95$). Maximum-Likelihood topologies were constructed using haplotypes for each individual nuclear locus. The phylogeny generated for Met-II, the most polymorphic target, is given as an example above. The most appropriate nucleotide substitution model was TrNef+G (three substitution rate categories) based on the AIC. Statistical support for major clades is given as equivalent bootstraps and posterior probabilities from consensus Maximum-Likelihood (1,000 pseudo-replicates) and Bayesian trees (based on the HKY+G model), respectively. Branch colors indicate isolate DTU (TcII, TcIII, TcV or TcVI). Colombian strain labels are underlined.
